# Supplementary material for: Automated Cell Lineage Reconstruction using Label-Free 4D Microscopy
Source: bioRxiv. 2024 Jan 22:2024.01.20.576449. Preprint. [Version 1] doi: 10.1101/2024.01.20.576449 (PMC10849476; doi:10.1101/2024.01.20.576449)
Supplement: Supplement 1 [file NIHPP2024.01.20.576449v1-supplement-1.pdf]

bioRxiv preprint doi: https://doi.org/10.1101/2024.01.20.576449; this version posted January 22, 2024. The copyright holder for this preprint (which was not certified by peer review) is the author/funder, who has granted bioRxiv a license to display the preprint in perpetuity. It is made available under aCC-BY 4.0 International license.

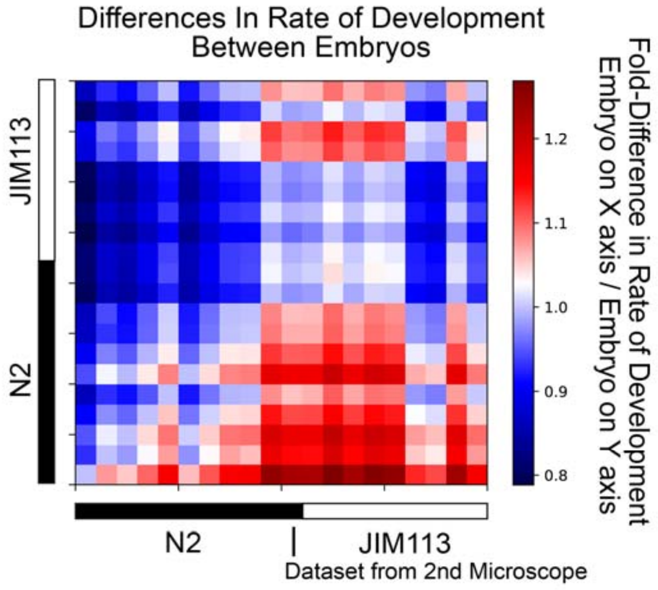

Figure S1. Comparison of global rate of development between all N2 (black bar) and JIM113 (white bar) embryos calculated pairwise using the first principal component as a non-parametric estimate of scaling between embryos. Each square represents a single pairwise comparison where the color reflects the fold-difference in cell cycle durations of the embryo indicated by the x-coordinate of the square relative to the embryo indicated by the y-coordinate of the square.

bioRxiv preprint doi: https://doi.org/10.1101/2024.01.20.576449; this version posted January 22, 2024. The copyright holder for this preprint (which was not certified by peer review) is the author/funder, who has granted bioRxiv a license to display the preprint in perpetuity. It is made available under aCC-BY 4.0 International license.

Supplemental Table 1. Cell Detection Performance on Fluorescence Images

Fluorescence	TP	FP	FN	Accuracy	Precision	Recall
s1_emb2	20431	416	175	0.972	0.980	0.992
s1_emb3	16211	620	127	0.956	0.963	0.992
s2_emb1	16502	504	72	0.966	0.970	0.996
s2_emb3	20755	328	200	0.975	0.984	0.990

Supplemental Table 2. Cell Detection Performance on embGAN-processed DIC Images

embGAN	TP	FP	FN	Accuracy	Precision	Recall
s1_emb2	21239	368	760	0.95	0.983	0.965
s1_emb3	16113	187	673	0.95	0.989	0.960
s2_emb1	18271	466	617	0.944	0.975	0.967
s2_emb3	21358	467	773	0.945	0.979	0.965

Supplemental Table 3. Cell Tracking Performance on Fluorescence Images

Fluorescence	TP	FP	FN(missing)	FN(incorrect)	FN	Accuracy	Precision	Recall
s1_emb2	20635	464	176	78	254	0.967	0.978	0.987
s1_emb3	16407	640	128	48	176	0.953	0.962	0.989
s2_emb1	16734	529	76	37	113	0.963	0.969	0.993
s2_emb3	20935	390	270	200	470	0.961	0.982	0.978

Supplemental Table 4. Cell Tracking Performance on embGAN-Processed DIC Images

embGAN	TP	FP	FN(missing)	FN(incorrect)	FN	Accuracy	Precision	Recall
s1_emb2	20693	730	766	440	1206	0.914	0.966	0.945
s1_emb3	15830	416	682	294	976	0.920	0.974	0.942

bioRxiv preprint doi: https://doi.org/10.1101/2024.01.20.576449; this version posted January 22, 2024. The copyright holder for this preprint (which was not certified by peer review) is the author/funder, who has granted bioRxiv a license to display the preprint in perpetuity. It is made available under aCC-BY 4.0 International license.

s2_emb1	17475	923	633	481	1114	0.896	0.945	0.940
s2_emb3	20946	754	786	384	1170	0.916	0.95	0.947
